# Supplementary material for: Multi-layered maps of neuropil with segmentation-guided contrastive learning
Source: Nat Methods. 2023 Nov 20;20(12):2011–20. doi: 10.1038/s41592-023-02059-8 (PMC10703674; doi:10.1038/s41592-023-02059-8)
Supplement: Supplementary file 1 — Supplementary Tables 1–3. [file 41592_2023_2059_MOESM1_ESM.pdf]

nature methods

Article https://doi.org/10.1038/s41592-023-02059-8

Multi-layered maps of neuropil with segmentation-guided contrastive learning

In the format provided by the authors and unedited

dataset	subcompartment	#training segments	total #training examples
human	axon	1,256	262,395
	dendrite	933	1,151,175
	soma	993	40,649
	astrocytic process	212	1,392,702
mouse	axon	121	4,653,326
	dendrite		2,343,169
	soma		13,830

Supplementary Table 1. Cellular subcompartment ground truth label sets.
The number of distinct segments and total number of training examples of each type available for subcompartment classification (Fig. 2). For both fully supervised training on the human dataset and linear classifiers trained on SegCLR embeddings, both full and reduced subsets of the training set were evaluated (Fig. 2d).

dataset	cell supertype	cell subtype and abbreviation	#segments	#embeddings total
human	neuron	Excitatory (E)	159	863,644
		Inhibitory (I)	52	91,283
	glia	Microglia cell (MGC)	36	29,536
		Oligodendrocyte precursor cell (OPC)	19	23,614
		Oligodendrocyte glia cell (OGC)	17	4,449
		Astrocyte (AC)	45	1,394,056
mouse	neuron	Layer 2/3 Pyramidal cell (P23)	95	1,281,244
		Layer 4 Pyramidal cell (P4)	23	254,156
		Layer 5 Pyramidal cell (P5)	36	633,328
		Layer 6 Pyramidal cell (P6)	8	67,504
		Basket cell (BC)	76	1,347,142
		Bipolar cell (BPC)	33	141,179
		Martinotti cell (MC)	30	460,301
		Neurogliaform cell (NGC)	17	126,407
		Thalamocortical axon (THLC)	11	108,639
	glia	Microglia cell (MGC)	9	22,819
		Oligodendrocyte precursor cell (OPC)	6	16,404
		Oligodendrocyte glia cell (OGC)	28	20,142
		Astrocyte (AC)	6	269,367

Supplementary Table 2. Cell type ground truth label sets.
The number of distinct segments and total number of embeddings available for cell type classification (Figs. 3-6). Note that only a fraction of the embedding nodes were used during training.

		Axon			Dendrite			Soma			Astrocyte		
Sample	F1	Precision	Recall	F1	Precision	Recall	F1	Precision	Recall	F1	Precision	Recall	F1
10	0.918	0.940	0.939	0.934	0.928	0.939	0.926	0.874	0.830	0.845	0.961	0.973	0.966
18	0.916	0.943	0.934	0.933	0.921	0.946	0.929	0.856	0.864	0.851	0.982	0.928	0.951
33	0.956	0.963	0.976	0.969	0.930	0.983	0.955	0.961	0.876	0.915	0.978	0.991	0.984
61	0.967	0.968	0.985	0.976	0.949	0.974	0.961	0.965	0.917	0.939	0.990	0.993	0.991
112	0.972	0.970	0.984	0.977	0.952	0.989	0.970	0.978	0.921	0.949	0.992	0.996	0.994
206	0.983	0.972	0.993	0.982	0.976	0.992	0.984	0.991	0.949	0.969	0.994	0.997	0.996
379	0.990	0.986	0.995	0.991	0.986	0.990	0.988	0.991	0.977	0.984	0.997	0.997	0.997
550	0.990	0.986	0.996	0.991	0.985	0.991	0.988	0.993	0.978	0.985	0.998	0.998	0.998
695	0.991	0.987	0.996	0.992	0.986	0.993	0.990	0.995	0.978	0.987	0.998	0.998	0.998
1274	0.994	0.992	0.996	0.994	0.991	0.994	0.993	0.994	0.989	0.992	0.999	0.998	0.999
2335	0.995	0.993	0.996	0.994	0.993	0.995	0.994	0.996	0.992	0.994	0.999	0.999	0.999
4281	0.996	0.994	0.996	0.995	0.995	0.996	0.995	0.996	0.994	0.995	0.999	0.999	0.999
7847	0.996	0.994	0.997	0.996	0.994	0.997	0.995	0.998	0.993	0.995	1.000	0.999	0.999
14384	0.997	0.995	0.997	0.996	0.997	0.996	0.997	0.997	0.995	0.996	0.998	0.999	0.999
26366	0.997	0.995	0.997	0.996	0.997	0.997	0.997	0.997	0.996	0.997	0.999	0.999	0.999
48329	0.997	0.995	0.998	0.996	0.996	0.997	0.997	0.998	0.994	0.996	0.998	0.999	0.999
88586	0.997	0.995	0.998	0.996	0.996	0.996	0.996	0.998	0.995	0.997	0.999	0.999	0.999
162377	0.997	0.996	0.997	0.997	0.996	0.996	0.996	0.998	0.994	0.996	0.998	0.999	0.999
297635	0.997	0.996	0.998	0.997	0.997	0.997	0.997	0.998	0.996	0.997	0.998	0.999	0.999
545559	0.997	0.996	0.998	0.997	0.997	0.997	0.997	0.998	0.996	0.997	0.999	0.999	0.999
1000000	0.997	0.996	0.998	0.997	0.996	0.996	0.996	0.998	0.995	0.997	0.999	0.999	0.999

Supplementary Table 3. Performance of a linear classifier trained on SegCLR embeddings for producing subcompartment labels on the h01 dataset.
Performances are means across multiple runs for sample sizes < 5,000. These data correspond to the black line in Figure 2d.
